# Analysis of lesion localisation at colonoscopy: outcomes from a multi-centre U.K. study

**DOI:** 10.1007/s00464-016-5313-z

**Published:** 2016-11-08

**Authors:** Susan J. Moug, Spyridon Fountas, Mark S. Johnstone, Adam S. Bryce, Andrew Renwick, Lindsey J. Chisholm, Kathryn McCarthy, Amy Hung, Robert H. Diament, John R. McGregor, Myo Khine, James D. Saldanha, Khurram Khan, Graham Mackay, E. Fiona Leitch, Ruth F. McKee, John H. Anderson, Ben Griffiths, Alan Horgan, Sonia Lockwood, Carly Bisset, Richard Molloy, Mark Vella

**Affiliations:** 1 Department of Surgery, Royal Alexandra Hospital, Corsebar Road, Paisley, PA2 9PN Scotland, UK; 2 Undergraduate Medical School, University of Glasgow, Glasgow, UK; 3 Department of Surgery, North Bristol NHS Trust, Bristol, UK; 4 Department of Surgery, University Hospital Crosshouse, Kilmarnock, UK; 5 Department of Surgery, Hairmyres Hospital, Lanarkshire, UK; 6 Department of Surgery, Glasgow Royal Infirmary, Glasgow, UK; 7 Department of Surgery, Royal Victoria Infirmary and Freeman Hospitals, Newcastle, UK; 8 Queen Elizabeth University Hospital, Glasgow, UK

**Keywords:** Colonoscopy, Lesion localisation, Multi-centred audit

## Abstract

**Background:**

Colonoscopy is currently the gold standard for detection of colorectal lesions, but may be limited in anatomically localising lesions. This audit aimed to determine the accuracy of colonoscopy lesion localisation, any subsequent changes in surgical management and any potentially influencing factors.

**Methods:**

Patients undergoing colonoscopy prior to elective curative surgery for colorectal lesion/s were included from 8 registered U.K. sites (2012–2014). Three sets of data were recorded: patient factors (age, sex, BMI, screener vs. symptomatic, previous abdominal surgery); colonoscopy factors (caecal intubation, scope guide used, colonoscopist accreditation) and imaging modality. Lesion localisation was standardised with intra-operative location taken as the gold standard. Changes to surgical management were recorded.

**Results:**

364 cases were included; majority of lesions were colonic, solitary, malignant and in symptomatic referrals. 82% patients had their lesion/s correctly located at colonoscopy. Pre-operative CT visualised lesion/s in only 73% of cases with a reduction in screening patients (64 vs. 77%; *p* = 0.008). 5.2% incorrectly located cases at colonoscopy underwent altered surgical management, including conversion to open. Univariate analysis found colonoscopy accreditation, scope guide use, incomplete colonoscopy and previous abdominal surgery significantly influenced lesion localisation. On multi-variate analysis, caecal intubation and scope guide use remained significant (HR 0.35, 0.20–0.60 95% CI and 0.47; 0.25–0.88, respectively).

**Conclusion:**

Lesion localisation at colonoscopy is incorrect in 18% of cases leading to potentially significant surgical management alterations. As part of accreditation, colonoscopists need lesion localisation training and awareness of when inaccuracies can occur.

Colonoscopy is currently the gold standard for detection of colorectal lesions and is recommended in the surveillance of colorectal cancers and higher risk colorectal lesions [1]. Colonoscopy also has another critical role where, in combination with radiological imaging, colonoscopy anatomically localises lesions, allowing optimal pre-operative surgical planning that is beneficial for both surgeon and patient. Recent work has suggested that the role of accurate colonoscopic lesion localisation has become increasingly important in the modern colorectal era for two reasons [2]. First, the establishment of the NHS Bowel Cancer Screening Programme (NHSBCSP) has led to the detection of earlier, and subsequently smaller, lesions that may not be visible on CT imaging [3]. Second, laparoscopic surgery with its reduced tactility, particularly with smaller lesions, is increasingly being offered to patients [4].

Previous publications have varied in the reported accuracy of colonoscopy with incorrect lesion localisation documented from 1.7 to 40.3% [5–20]. The majority of these studies are retrospective and single centre in design, making conclusions difficult. A recent small prospective multi-centre audit in the West of Scotland reported incorrect lesion localisation at colonoscopy in 19% of cases that led to an on-table alteration in surgical management in 6% [2]. Furthermore, the only factor found to be influencing accurate lesion localisation was incomplete colonoscopy.

This study aimed to perform a large multi-centre audit across the U.K. to first assess the accuracy of colonoscopic lesion localisation and any subsequent operative consequences and second to determine potential influencing factors.

## Methods and patients

Pilot data were collected prospectively from October 2011 to April 2012 and then October 2012 to April 2013, in hospital sites in Greater Glasgow and Clyde and NHS Ayrshire and Arran. U.K. data collection started September 2013 to October 2014 from a total of 8 centres: Hairmyres General, East Kilbride, NHS Lanarkshire; Royal Alexandra Hospital, Paisley, Western Infirmary, Glasgow and Glasgow Royal Infirmary, all NHS Greater Glasgow and Clyde; Crosshouse Hospital, Kilmarnock, NHS Ayrshire and Arran; Freeman Hospital and Royal Victoria infirmary, both Newcastle Hospitals NHS Foundation Trust and Frenchay Hospital, North Bristol NHS Trust.

The ALLaC study (Accurate Lesion Localisation at Colonoscopy) was registered with Clinical Effectiveness Unit, Greater Glasgow and Clyde. Each participating site registered locally as per local policies and procedures.

Any patient undergoing elective curative surgical resection for a benign or malignant colorectal lesion/s that had undergone a colonoscopy prior to surgery was included. All patients had undergone colonoscopy either because of a positive faecal occult blood test through the NHS Bowel Cancer Screening Programme (‘screener’) or because they had been referred from primary care with colorectal symptoms (‘symptomatic’). The local investigator at each participating centre identified patients from departmental multi-disciplinary colorectal cancer meetings and/or departmental operating lists, and the proforma was completed within 24 h of the patient undergoing surgery (“Appendix”). Patients who had undergone neo-adjuvant chemo-radiotherapy as primary treatment or who underwent palliative surgery were excluded from this study.

The study proforma recorded patient, colonoscopic and imaging factors. Patient factors included: age, sex, BMI (body mass index), type of referral (screener or symptomatic) and previous abdominal surgery. Colonoscopic factors recorded were: caecal intubation [defined as ‘passage of the scope beyond the ileocaecal valve into the caecal pole or terminal ileum (or anastomosis)’ confirmed on electronic colonoscopy report] [21]; use of endoscopic scope guide; reasons for incomplete scope; experience/accreditation of the colonoscopist (Joint Advisory Group Accreditation awarded); tattoo placed and lesion localisation [22]. Imaging factors documented were: modality of imaging used and lesion localisation.

Lesion localisation was standardised into nine segments (caecum, ascending, hepatic flexure, transverse, splenic flexure, descending, sigmoid, rectum and ‘other’) with true lesion location defined as the intra-operative surgical location to allow comparison with colonoscopic and imaging localisation. Any changes to planned surgical management as a result of an alteration in lesion localisation intra-operatively were recorded.

### Statistics

All categorical variables were analysed with the Chi-squared test with 95% confidence intervals reported where appropriate. SPSS software (v22.0, Chicago, Illinois, USA) was used, and *p* values of less than 5% were taken as significant.

## Results

365 forms were submitted for patients that had undergone colonoscopy followed by surgical resection in the eight centres across the U.K. On review of the forms, one case was excluded due to incomplete information in multiple sections.

### Patient factors (Table 1)

The mean age of the study population was 67.5 years (27–90 range). There were slightly more males (54%), with 75% being at least overweight and 39% recorded as obese. The majority of lesions found were in the colon, solitary, malignant and in patients referred with symptoms. 51% of the population had undergone previous abdominal surgery.

**Table 1:** Demographics and description of patients undergoing colonoscopy and the lesions found

Total number of patients	364	
Age at scope (mean)	67.5	Range 27–90 years
Male/female	198:166	54:46%
BMI^a^		
Mean	28.3	Range 15–48 kg/m^2^
<20	14 (5%)	
20–24.9	55 (20%)	
25.0–30	99 (36%)	
>30	105 (39%)	
Screening: symptomatic^a^	112:250	31:69%
Previous abdominal surgery: yes:no^a^	181:176	51:49%
No of lesions found 1:>1^a^	340:23	94:6%
Malignant lesion:benign lesion	318:46	87:13%
Colonic lesion:rectal lesion	262:102	72:28%

^a^Missing cases: BMI *n* = 91; Screening versus symptomatic *n* = 2; previous abdominal surgery *n* = 7; number of lesions found *n* = 1

### Colonoscopy factors (Table 2)

The majority of colonoscopists were accredited (60%), but only 36% used a scope guide. Only 36% tattooed a lesion’s location. There was no clear pattern to why certain lesions were tattooed and others not, as the tattooing was performed throughout the nine segments of the colon. Caecal intubation was reported in 73% of cases, with reasons reported for failure as: obstructing lesion 68%, poor bowel preparation leading to lesion not seen 2%, looping sigmoid/colon preventing advancement 15% and not reported 15%. Except for the obstructing lesions, all incomplete colonoscopies went on to have further imaging by CT pneumocolon.

**Table 2:** Description of colonoscopic factors

Total number of patients	364	
Accreditation yes:no^a^	217:145	60:40%
Scope guide yes:no^a^	125:224	36:64%
Tattoo yes:no^a^	132:228	37:63%
Caecal intubation yes:no	267:97	73:27%

^a^Missing cases: accreditation *n* = 2; scope guide *n* = 15; tattoo *n* = 4

### Colonoscopic lesion localisation (Table 3)

Table 3 shows the true anatomical location of the colorectal lesions at surgery, with the majority being colonic. The lesions are distributed throughout the nine segments but are mainly in the sigmoid (22.3%), rectum (28%) and right colon (32.7%). There were small non-significant variations between surgical and colonoscopic localisation in most of the segments. However, colonoscopy significantly underestimated the number of rectal lesions recorded (77 vs. 102) and significantly overestimated the number of sigmoid lesions (112 vs. 81).

**Table 3:** Comparison of segments of lesion localisation at colonoscopy versus surgery

Anatomical location	Colonoscopy	Surgery	Difference	Chi square
	*n* (%)	*n* (%)	*n*	
Total colonic	287 (79)	262 (72)		0.008*
Total rectal	77 (21)	102 (28)		0.045*
Caecum	68 (18.7)	70 (19.2)	2	0.093
Ascending colon	50 (13.7)	49 (13.5)	1	0.132
Hepatic flexure	15 (4.1)	10 (2.7)	5	0.133
Transverse colon	19 (5.2)	23 (6.3)	4	0.191
Splenic flexure	13 (3.6)	18 (4.9)	5	0.227
Descending colon	9 (2.5)	10 (2.7)	1	0.163
Sigmoid colon	112 (30.8)	81 (22.3)	31	0.025*
Rectum	77 (21.1)	102 (28)	25	0.079
Other^a^ (i.e., anastomosis)	1 (0.3)	1 (0.3)	0	n/a

Bold values indicate *p* < 0.05

* *p* < 0.05 is level of significance

^a^These are included in this group, but colonoscopy localisation was incorrect

Overall, two hundred and ninety-nine patients had their lesion/s located within the correct segment at colonoscopy (82%).

### Imaging lesion localisation and factors (Table 4)

All patients underwent a pre-operative CT (either abdomen/pelvis or CT colonography). Pre-operative CT visualised a colorectal lesion/s in only 73% of cases (265/361) with a significant reduction in visualising lesion/s in screening patients (64% screener vs. 77% in symptomatic; *p* = 0.008). Accurate localisation of lesion/s was reported in 213 cases, leading overall accuracy of CT to be 59%. Including only the cases where the lesion/s could be seen on CT, the accuracy increased to 80% (213/265).

**Table 4:** Description of imaging factors

Total number of patients	364	
CT performed pre-operatively yes:no^a^	363:1	99.7:0.3%
CT lesion seen yes:no^b^	265:96	73:27%
CT lesion correctly localised lesion/s yes:no	213:52	80:20%
MRI performed pre-operatively yes:no	75:289	21:79%
MRI lesion seen yes:no^b^	65:9	88:12%
MRI lesion correctly localised lesion/s yes:no	64:1	98:2%

^a^CT included *n* = 2 CT colons, remainder contrast enhanced CT abdomen/pelvis

^b^Missing cases: CT lesion seen *n* = 3; MRI lesion seen *n* = 1

Combining CT and colonoscopy localisation correctly localised the lesion in 87.1% of cases.

Only 21% underwent pre-operative MRI (*n* = 75) potentially reflecting the number of rectal lesions located at colonoscopy (*n* = 77). Of those cases, a lesion/s was localised in 65, with 64 cases being accurate. Overall lesion localisation for MRI was 88% (65/74 as one case localisation missing).

#### Changes to intra-operative management

Of the total number of lesion/s being incorrectly localised at colonoscopy (*n* = 65), 19 cases underwent a change in intra-operative management (5.2% of all cases) (Table 5). The majority of these changes were a result of a lesion being incorrectly localised to the segment more proximal or distal at colonoscopy, with surgical management adapting accordingly. The majority of these cases were open procedures (*n* = 12) with 3 of the 7 laparoscopic cases being converted to open and 1 laparoscopic case performing an open rectal dissection, the later due to the tumour being more distal than thought pre-operatively.

**Table 5:** Changes in planned surgical management due to altered intra-operative lesion localisation

Planned	Actual	Reason	No of cases	Lap versus open^a^
Right hemicolectomy	Extended right hemicolectomy	Lesion in transverse colon rather than ascending colon	4	x2 open, x2 con
	Subtotal colectomy	Lesion not caecal, but transverse with erosion into middle colics	1	X1 con
Anterior resection	Left hemicolectomy	Lesion in descending, not sigmoid	3	X1 lap; x2 open
	Full TME	Sigmoid to low-rectum	5	X3 open; x1 lap; x1 lap assisted
Left hemicolectomy	Anterior resection	Descending actually distal sigmoid	1	X1 lap
Sigmoid colectomy	Left hemicolectomy	Descending lesion rather than sigmoid	1	X1 open
	Anterior resection	Rectal lesion, not sigmoid, full TME required.	2	X2 open
Ileocolic anastomosis	Subtotal colectomy	Not anastomotic recurrence, but metachronous locally advanced sigmoid cancer	1	X1 open
Extended right hemicolectomy	Subtotal colectomy	Splenic flexure lesion, not transverse	1	X1 open

^a^Open means the case was started and completed open; lap means completed laparoscopically; lap assisted means rectal dissection converted to open; con means converted from lap to open

Further difficulties reported during these 19 altered surgical management cases included impalpable lesions or inappropriate/absent tattooing. Strategies employed to overcome these difficulties were: on-table colonoscopy with or without colorectal lavage; insertion of hand ports to allow palpation and accurate small lesion localisation; further port/s insertion to accommodate for alteration in surgical resection.

### Factors influencing accurate lesion localisation at colonoscopy (Table 6)

Analysis of patient and colonoscopic factors found that colonoscopy accreditation, use of the scope guide, caecal intubation and previous abdominal surgery all significantly influenced accurate lesion localisation. On multi-variate analysis, both caecal intubation and use of the scope guide remained significant (HR 0.35, 0.20–0.60 95% CI and 0.47; 0.25–0.88, respectively).

**Table 6:** Univariate analysis of potential influencing patient and colonoscopic factors on accurate lesion localisation at colonoscopy

	Total patients *N* = 364	Colonoscopic lesion localisation		
		Accurate *N* = 299	Inaccurate *N* = 65	*p* value (95% CI)
Sex				
Male	198	167	31	0.231
Female	166	312	34	
Age at scope				
<65	146	119	27	0.795
>65	218	180	38	
Referral				
Symptomatic	250	205	45	0.811
Screening	112	93	19	
Abdominal surgery				
Yes	181	141	40	0.037*
No	176	152	24	(0.69–2.41)
Caecal intubation				
Yes	267	233	34	<0.001*
No	97	66	31	(0.20–0.60)
Scope guide used				
Yes	125	111	14	0.034*
No	244	179	45	(0.26–0.91)
Tattoo				
Yes	132	106	26	0.270
No	228	189	39	
BMI				
<20	14	10	4	0.409
20–25	55	44	11	
25–29.9	99	84	15	
>30	105	91	14	
Accreditation				
Yes	217	186	31	0.038*
No	145	112	33	(0.47–1.60)

## Discussion

This prospective multi-centred U.K. study has found colonoscopy to incorrectly localise colorectal lesions in 18% of cases, leading to altered surgical management in theatre in 5.2% of cases. Combining the locations of colonoscopy and CT increased correct lesion localisation to 87.1%. However, this study has confirmed that CT imaging does not visualise lesions in over a quarter of cases, particularly in the screening population. With earlier and smaller lesions expected to continue to be detected with NHSBCSP, the role of colonoscopy in optimal pre-operative surgical planning is likely to become increasingly important in the modern era.

Previous publications have reported significant variability in the accuracy of colonoscopy to localise lesions, from 59.7 to 98.3% [5–20]. In a previous publication from this group, Bryce et al. [2] reviewed the literature highlighting many of these studies have been retrospective, small in number, single centre or single endoscopist in design. With this heterogeneity, several influencing factors have been proposed to influence lesion localisation: increasing age; previous abdominal surgery and incomplete colonoscopy [5, 8, 14]. This is in comparison with this current work that has encompassed eight U.K. hospital sites with over fifty different colonoscopists, several of whom would have been blinded as the data were recorded peri-operatively, not at the time of the colonoscopy. Furthermore, the larger number of patients in this study allows for further statistical analysis of influencing factors, with use of the scope guide, colonoscopic accreditation, caecal intubation and previous abdominal surgery all shown to be significant.

The scope guide is regarded by many colonoscopists as a teaching aid and is not always routinely available. Indeed the guidelines for JAG accreditation [22] state that as part of achieving accreditation, use of the scope guide is not mandatory. Results from this audit suggest that this approach needs to be changed as routine use of the scope guide could educate colonoscopists leading to improved lesion localisation and as a result should be available on all screening and symptomatic colonoscopy lists.

Experience of the colonoscopist was also significant with those not JAG accredited having greater inaccuracies of localising lesions. Currently, only JAG accredited colonoscopists can perform screening lists independently and perhaps this criterion should be applied to symptomatic referrals so that in the event of diagnosis of a lesion requiring surgical resection optimal localisation can occur [22]. In addition, it is worth highlighting that to achieve accreditation, colonoscopists must achieve competence in four domains: assessment; consent and communication; endoscopic skills and diagnostic and therapeutic ability [23]. Within these domains, focused training on localisation could be made mandatory.

Education would be the first step as many, particularly non-surgeons, may not be aware of the difficulties for both patient and surgeon that incorrect localisation and/or inappropriate tattooing can cause. Included in this approach could be the proposal that all recto-sigmoid lesions undergo rigid proctoscopy to confirm tumour height as an incorrectly located rectal tumour that alters abdominal surgery to pelvic surgery has major implications for the patient (higher anastomotic leak risk and urinary and sexual dysfunction). These changes could be supported by the introduction of specific and mandatory questions on the electronic colonoscopy record about lesion localisation: tattoo yes/no; tattoo sites (with proximal and distal options suggested depending on lesion site); rigid proctoscopy performed yes/no. Currently, it is optional for the colonoscopist to include these pieces of information which may partly explain the low number of lesions tattooed in this study (36%).

Incomplete colonoscopy means that the colonoscopist has less visual exposure to the landmarks that they are trained to recognise and the complete colonoscopy allows two views (insertion and withdrawal) to increase the probability of correctly localising a lesion. The two main reasons for incomplete colonoscopy were obstructing lesions and sigmoid loops and, especially if the scope guide is not used, one can see why the colonoscopist could become disorientated in this situation.

Previous abdominal surgery included all types, not just colorectal resections, and the experienced colonoscopist is aware that previous surgery can make gentle passage of the scope difficult, particularly in women who have undergone a hysterectomy. However, colonoscopists and surgeons must now be aware that in this same group of patients, localisation can also be difficult. Only a small number of patients in this audit had undergone a resection of the colon or rectum, making conclusions about lesion localisation in this specific population limited; however, one extreme case is presented in this study where an anastomotic recurrence was not correctly reported leading to a surgical alteration in management.

There was only a 5.2% change in on-table management due to inaccurate lesion localisation at colonoscopy. This figure would have been higher if not for CT in combination with colonoscopy, increasing the number of correctly localised lesions (to 87.1%). These incorrect localisations mainly occurred at a more proximal or distal segment with no change in management. However, when a change in management occurred (29% of inaccurate cases), it was significant with 3 laparoscopic cases converted to open and one requiring an unexpected open TME dissection. This raises the possibility that incorrect lesion localisation could make laparoscopic surgery vulnerable to on-table alterations.

From a surgical viewpoint, these results demonstrate key areas where incorrectly pre-operative localisation can have significant operative changes: hepatic and transverse colon (decision to take middle colics and further dissection for an extended right hemicolectomy); splenic flexure (extended right hemicolectomy vs. left hemicolectomy); sigmoid versus rectum (to perform a total mesorectal excision/covering ileostomy and consideration to taking down the splenic flexure). It is beyond the remit of this work to document what the short- and long-term implications for the patients with altered management were; however, with the additions of on-table colonoscopy/lavage, further dissection and extra ports, it is unsurprising that many proformas made the comment ‘increased operating time’.

## Limitations

This is not a consecutive series of patients, so case selection bias may be present. Identification of the patients was left to the local investigator who may have included or excluded patients for various reasons. Excluding the authors, the majority of the endoscopists were blinded to this study. The surgeon and radiologist were not blinded to the result of the lesion localisation at colonoscopy, leading to verification bias. This limitation, however, reflects the pragmatic nature of the study and current clinical practice.

## Conclusion

Surgical planning pre-operatively is becoming increasingly reliant on accurate lesion localisation at colonoscopy. Colonoscopists need to be educated of the key anatomical areas where inaccuracies occur and have increased vigilance where caecal intubation has been unsuccessful. Routine clinical use of the scope guide could potentially increase correct localisation, minimising on-table alterations in surgical management and optimising outcomes for surgeons and patients.
